# Mind the Motion: Feasibility and Effects of a Qigong Intervention on Interoception and Well-Being in Young Adults

**DOI:** 10.3390/healthcare14020202

**Published:** 2026-01-13

**Authors:** Rebecca Ciacchini, Alessandro Lazzarelli, Giorgia Papini, Aleandra Viti, Francesca Scafuto, Graziella Orrù, Angelo Gemignani, Ciro Conversano

**Affiliations:** 1 Department of Surgical, Medical, Molecular Pathology and Critical Care Medicine, University of Pisa, 56126 Pisa, Italy; giorgia.papini@med.unipi.it (G.P.); aleandra.viti@ing.unipi.it (A.V.); francesca.scafuto80@gmail.com (F.S.); graziella.orru@unipi.it (G.O.); angelo.gemignani@unipi.it (A.G.); ciro.conversano@unipi.it (C.C.); 2 School of Advanced Studies, University of Camerino, 62032 Camerino, Italy; 3 Department of Civilizations and Forms of Knowledge, University of Pisa, 56126 Pisa, Italy; a.lazzarelli@gmail.com

**Keywords:** Qigong, interoception, emotion regulation, mindfulness, contemplative practice, university students, young adults, psychological well-being, mind-body interventions

## Abstract

**Highlights:**

**What are the main findings?**

- A 12-week Qigong program significantly improved multiple dimensions of interoceptive awareness (e.g., Body Listening, Trusting, Self-Regulation, Emotional Awareness) and enhanced emotion regulation, mindfulness, and psychological well-being among university students.
- Participants with lower baseline interoceptive ability or higher trait anxiety benefited the most, showing greater emotional awareness and self-regulatory capacity.

**What are the implications of the main findings?**

- Qigong, by integrating both top-down and bottom-up processes, may represent an effective, low-cost, and replicable mind–body intervention for promoting mental health and resilience in academic settings.
- The results support the inclusion of interoceptive and movement-based practices in preventive psychological programs, emphasizing their role in enhancing emotional regulation and stress adaptation among young adults.

**Abstract:**

**Background/Objectives**: The present exploratory study evaluates the feasibility and psychological effects of a structured Qigong intervention implemented in an Italian university setting. Qigong is a traditional Chinese mind–body practice combining gentle movements, breathwork, and mindful attention, aimed at enhancing mind–body integration and interoceptive awareness. **Methods**: A total of 332 undergraduate students voluntarily enrolled in a 12-week Qigong program. The intervention was based on Neidan Qigong and integrated both static and dynamic exercises. Psychological functioning was assessed through several self-report measures evaluating a range of constructs, including mindfulness (FFMQ), interoceptive ability (MAIA), perceived stress (PSS), depression, anxiety, and stress (BDI; DASS-21; STAI Y), emotion regulation (DERS), alexithymia (TAS), and sleep quality (PSQI). **Results**: A total of 114 students completed the intervention. The protocol was well received by participants and demonstrated high feasibility in the academic context, with good attendance rates and overall engagement. Preliminary findings indicate consistent improvements across several psychological domains. **Conclusions**: The results suggest that Qigong may be associated with improvements in mental health and well-being in young adults and may represent a promising, low-cost intervention. The findings should be interpreted as preliminary. Further research using controlled and methodologically rigorous designs is needed to assess the stability of these effects over time, incorporate physiological measures, and clarify the specific therapeutic contribution of spontaneous movement within Qigong practice.

## 1. Introduction

### 1.1. Qigong: Characteristics and Practice

Qigong is an umbrella term referring to a group of traditional Chinese mind–body practices rooted in Taoist philosophy and Traditional Chinese Medicine [1]. Historically consolidated within the Neidan (Internal Elixir) tradition, these practices emphasized internal regulation of bodily and mental processes rather than external or chemical alchemy [1]. During the twentieth century, particularly in the context of modernization reforms in China, Qigong practices were reframed within medical and scientific discourse and formally integrated into healthcare settings as preventive and therapeutic interventions [2]. Despite the heterogeneity of styles and schools, Qigong is characterized by the systematic integration of static and dynamic exercises, combining gentle, repetitive movements, postural regulation, breath control, and focused attention [3]. It is therefore commonly described as a movement-based embodied contemplative practice [3]. From a psychophysiological perspective, Qigong engages both voluntary, attention-driven processes and more spontaneous, involuntary bodily responses, which have been conceptualized as interacting top-down and bottom-up regulatory pathways [4]. The Neidan Qigong approach adopted in the present study explicitly integrates these dimensions. Participants are guided to cultivate awareness of bodily sensations through structured contemplative practices (e.g., body scanning, breath-focused attention), while also being encouraged to allow spontaneous movements to emerge without deliberate control [5]. Through this combination of intentional attention and embodied responsiveness, Qigong is hypothesized to influence psychological functioning by modulating proprioceptive and interoceptive processes. Proprioception refers to the perception of musculoskeletal position and movement [6] and is stimulated through balance, coordination, and postural training, which have been shown to influence mood and well-being [7]. Interoception, in contrast, involves the perception and integration of internal physiological signals into conscious experience [8], a capacity that may be enhanced through mind–body practices such as Qigong [9]. Additional historical and conceptual details on Qigong are provided in the Supplementary Materials.

### 1.2. Mental Health and Well-Being in University Students

University students represent a population at risk for psychological distress due to academic demands, performance pressure, social transitions, and uncertainty about the future [10]. Elevated levels of perceived stress, anxiety, depressive symptoms, and sleep disturbances are widely documented in this group and may interfere with both academic functioning and long-term mental health [10,11]. Difficulties in emotion regulation are particularly relevant, as they contribute to maladaptive stress responses and vulnerability to psychopathology [12,13,14,15].

Emotion regulation refers to the processes through which individuals monitor, evaluate, and modify emotional reactions in order to adapt to situational demands [12]. Deficits in these processes are transdiagnostic features of many psychological disorders and are closely linked to dysregulation of autonomic and neuroendocrine stress systems [16,17,18,19,20]. Conversely, effective emotion regulation is associated with resilience, psychological flexibility, and adaptive coping [14,15]. Among students, emotion regulation capacities have been shown to predict academic performance, stress tolerance, and overall well-being [21,22,23,24].

Interoceptive awareness plays a central role in emotion regulation, as the ability to perceive and interpret bodily signals supports emotional recognition and modulation [25,26,27,28]. Impairments in interoceptive processing have been associated with heightened anxiety, stress reactivity, alexithymia, and difficulties in adaptive coping [29,30,31,32]. Interventions that foster embodied awareness and self-regulation may therefore be particularly relevant in academic contexts, where psychological demands are high and preventive approaches are needed.

### 1.3. Evidence on Qigong Practice and Psychological Outcomes

A growing body of literature supports the psychophysical benefits of Qigong practice across different populations. Systematic reviews and meta-analyses of randomized controlled trials reported improvements in physical functioning, immune parameters, and psychological outcomes, including reductions in stress, anxiety, and depressive symptoms after a qigong-based intervention [33,34,35,36]. These effects have been observed using both self-report measures (e.g., DASS-21, STAI, PSS) and physiological markers such as cortisol and adrenocorticotropic hormone [37,38,39,40]. Qigong practice has also been shown to influence autonomic nervous system activity and hypothalamic–pituitary–adrenal axis functioning, with a particular promotion of parasympathetic activation and stress recovery [39,41,42]. These mechanisms are closely linked to emotion regulation and affective stability [16,17,18]. Improvements in emotional functioning have been reported not only in clinical populations (e.g., chronic illness, substance use disorders, trauma exposure) [43,44,45,46,47,48,49], but also in non-clinical samples, including college students [11,50,51,52].

Importantly, Qigong appears to be associated with enhanced interoceptive awareness, a process considered central to emotion regulation and psychological well-being [9,53,54]. Preliminary evidence indicates that Qigong practice is linked to increased bodily awareness, greater emotional clarity, and reduced alexithymic traits. Improvements in interoceptive awareness have, in turn, been associated with greater use of adaptive emotion regulation strategies, such as cognitive reappraisal, and with reduced stress reactivity [7,27,28,55,56]. In this context, it is important to distinguish interoceptive awareness from interoceptive accuracy: while the latter is typically assessed through objective performance-based tasks (e.g., heartbeat detection), interoceptive awareness refers to the subjective perception, appraisal, and regulatory use of bodily signals. Accordingly, the present study focuses specifically on interoceptive awareness, as conceptualized within multidimensional self-report frameworks [57].

Although these mechanisms have been extensively examined within other contemplative-based interventions such as, for example, mindfulness programs, their role in Qigong practice and efficacy remains comparatively underexplored, particularly in young, non-clinical populations [9].

### 1.4. Aims of the Present Study

Despite increasing interest in Qigong as a mind–body intervention, several gaps remain. First, few studies have systematically examined interoceptive awareness and emotion regulation as primary outcomes of Qigong practice, especially using multidimensional measures. Second, evidence in university student populations is still limited, despite their vulnerability to stress-related difficulties. Third, the specific contribution of practices integrating both voluntary attention and spontaneous movement has rarely been addressed.

The present study should be framed as an exploratory and feasibility-oriented investigation aimed at examining the psychological effects of a structured Qigong intervention implemented in a university setting. Analyses focused primarily on changes in interoceptive awareness, emotion regulation, mindfulness, perceived stress, and anxiety. Additional psychological domains, including depressive symptoms and related outcomes, were assessed to provide a broader dimensional characterization of change over time. In addition, the study explored whether baseline individual characteristics were associated with differential responsiveness to the intervention and evaluated the feasibility and acceptability of delivering Qigong within an academic context.

## 2. Materials and Methods

### 2.1. Participants and Study Design

The present study involved Italian university students from the University of Pisa (Department of Civilizations and Forms of Knowledge) who voluntarily chose to participate in a Qigong protocol offered as a workshop, presented as an optional component of the General Psychology course. Students received an additional point on their exam score (one out of thirty) for their participation. A total of 331 students completed the pre-intervention assessment. Of these, 199 completed the post-intervention assessment, and 114 completed both assessments and were therefore included in the final analysis. No specific inclusion or exclusion criteria were applied beyond voluntary participation. Attendance and home practice were recorded descriptively but were not used as exclusion criteria.

### 2.2. Procedure

Participants took part in a 3-month Qigong workshop, which included twelve in-person guided intervention sessions and a requested daily home practice. The in-person sessions took place in a university classroom and were led by author AL, with support from a second facilitator. Each session lasted one hour, while the home practice was expected to last 15 min per day.

The workshop followed a structured protocol based on Neidan Qigong practices (see Table S1, Supplementary Materials). General information about the protocol was provided at the beginning of the first session. Each session was designed to integrate both static and dynamic exercises, as well as top-down and bottom-up approaches. At least one exercise from each category was selected weekly for the home practice. Due to classroom space limitations, exercises were performed in either seated or standing positions. Each session concluded with a brief group discussion during which facilitators elicited participants’ feedback on the practice.

All in-person sessions followed a similar structure, divided into three main phases:

1. Opening Phase: Mindfulness awareness, body scanning with a focus on sensations, breathwork, and sound emission.
2. Central Phase (Repetitive Movements): Gentle, repetitive motions that open energy channels and joints including rolling shoulders forward and backward, turning the head in all directions, circling the wrists, lifting the heels while engaging the whole body, shaking arms and legs, body brushing, rotating the waist, stretching the knees and ankles, balanced oscillations, spiraling movements, body shaking, body swinging; Movements repeated for at least 10 min including body bouncing, jumping, and spontaneous movements.
3. Closing Phase: Standing meditation (Zhan Zhuang) and sitting meditation.

### 2.3. Measures

Participants provided demographic information (e.g., age, gender, marital status). They also reported their previous experience with meditation and Qi Gong, including the type of practice and frequency. At post-intervention, students evaluated their attendance, frequency of home practice, and overall interest in the course, and provided qualitative feedback through a brief set of open-ended questions exploring their subjective experience of the Qigong practice, including perceived benefits, difficulties encountered during the exercises, and suggestions for improvement. These questions were designed to capture experiential and process-related aspects not fully addressed by standardized questionnaires. The full list of questions is reported in the Supplementary Materials (Table S2).

Moreover, the assessment battery comprised the following questionnaires. As already stated, and in line with the exploratory design of the study and the extensive assessment battery, not all administered measures were entered into the primary quantitative analyses. Selected instruments were included for descriptive or exploratory aims and are accordingly reported in the Supplementary Materials or used in targeted analyses (e.g., dropout comparisons).

- Five Facet Mindfulness Questionnaire (FFMQ) [58,59]: A 39-item self-report measure assessing five mindfulness facets: observing, describing, acting with awareness, non-judging of inner experience, and non-reactivity to inner experience.
- Perceived Stress Scale (PSS) [60,61]: A 10-item questionnaire evaluating the degree to which situations in one’s life are appraised as stressful over the past month.
- Multidimensional Assessment of Interoceptive Awareness (MAIA) [62,63]: A 32-item scale measuring eight dimensions of interoceptive body awareness, including noticing, attention regulation, and trusting body sensations.
- Interpersonal Accuracy Scale (IAS)* [64]: A performance-based tool assessing the ability to accurately infer others’ emotional states from nonverbal cues.
- Interpersonal Competence Questionnaire (ICQ)* [65,66]: A 40-item self-report instrument evaluating five domains of social competence: initiation, negative assertion, disclosure, emotional support, and conflict management.
- Difficulties in Emotion Regulation Scale (DERS) [67,68]: A 36-item measure capturing six facets of emotion regulation difficulties: non-acceptance, goals, impulse, awareness, strategies, and clarity.
- Depression Anxiety Stress Scales—short form (DASS-21) [69,70]: A 21-item short version assessing severity of depression, anxiety, and stress symptoms over the past week.
- Toronto Alexithymia Scale (TAS) [71,72]: A 20-item scale evaluating three core features of alexithymia: difficulty identifying feelings, difficulty describing feelings, and externally oriented thinking.
- Pittsburgh Sleep Quality Index (PSQI)* [73,74]: A 19-item self-rated questionnaire assessing sleep quality and disturbances over the past month across seven components.
- Resilience Scale (RS)* [75,76]: A 25-item measure of psychological resilience, focusing on personal competence, acceptance of self and life, and adaptability.
- Self-Compassion Scale (SCS)* [77,78]: A 26-item scale assessing six components of self-compassion: self-kindness, self-judgment, common humanity, isolation, mindfulness, and over-identification.
- State-Trait Anxiety Inventory (STAI) [79,80]: A 40-item inventory distinguishing transient state anxiety (20 items) from stable trait anxiety (20 items).
- Beck Depression Inventory–II (BDI-II) [81,82]: A 21-item self-report questionnaire assessing the severity of depressive symptoms over the past two weeks. Higher scores indicate greater depressive symptomatology.
- Positive and Negative Affect Schedule (PANAS)* [83,84]: A self-report measure assessing positive and negative affective states through two independent subscales. Participants indicate the extent to which they experienced each affective state during the reference period.

* Measures marked with an asterisk were included for descriptive or exploratory purposes and were not entered into the primary quantitative analyses.

Adverse events were not systematically monitored through a predefined reporting protocol; however, facilitators continuously observed participants during sessions, and subjective experiences, including discomfort or anxiety, were collected through post-intervention open-ended feedback.

### 2.4. Dropout Rate

A total of 331 participants were initially enrolled in the study. Between the pre- and post-intervention assessments, there was a dropout rate of 65.66%, resulting in 114 participants who completed both assessments.

### 2.5. Ethical Considerations

Participation in the study was voluntary, and all participants provided informed consent. The study was approved by Pisa’s University Bioethics Committee [deliberates n.54/2024 on 27 September 2024, n. 63/2025 on 29 September 2025] and conducted in accordance with the Declaration of Helsinki.

### 2.6. Statistical Analyses

Analyses were performed using JASP (Version 0.19.3.0; JASP Team, Amsterdam, The Netherlands). Descriptive statistics were computed for all variables, and normality was assessed using the Shapiro–Wilk test. Paired samples *t*-tests were performed for normally distributed variables, while Wilcoxon signed-rank tests were used for non-normally distributed variables. Effect sizes were reported using Cohen’s d and rank-biserial correlation, respectively. Correlations between pre–post difference scores were computed to explore associations between improvements in different psychological domains. Pearson’s r was used for normally distributed difference scores, and Spearman’s ρ for non-normally distributed ones. Linear regression analyses were conducted to examine whether baseline measures predicted post-intervention change scores. Feasibility and acceptability were assessed through analysis of dropout rates and session attendance. Despite the reduced final sample size due to attrition, the statistical power was sufficient for the analysis.

## 3. Results

Consistent with the exploratory design, results are presented following a hierarchical analytic approach. Analyses focused primarily on a set of core outcomes—namely mindfulness, interoceptive awareness, emotion regulation, perceived stress, and anxiety—which constituted the focus of within-subject comparisons, correlational analyses, and graphical representations. Additional measures assessing related psychological domains (e.g., depressive symptoms, alexithymia, sleep quality) were treated as secondary outcomes and were included to provide a broader dimensional characterization of changes following the intervention; these variables were examined through pre–post comparisons but were not included in the correlational analyses. Finally, several further measures were administered for exploratory or descriptive purposes, allowing a preliminary evaluation of their appropriateness and descriptive value in this context, and were therefore not included in the primary quantitative analyses, as they were not central to the study objectives.

### 3.1. Feasibility, Sociodemographic Characteristics, and Qualitative Feedback

The final sample consisted of 114 students who completed both pre- and post-intervention evaluations. Participants had a mean age of 20.97 years (SD = 4.76; range = 18–62), and the majority identified as female (75.4%), followed by male students (22.8%), with a small proportion identifying outside the binary gender categories (1.75%). Only 29.8% reported prior meditation experience, and 2.6% had previously practiced Qigong, indicating that most participants were novices to contemplative movement-based practices.

The intervention demonstrated good feasibility and acceptability. Students reported a mean attendance of 79.1% (SD = 16.6), rated their interest at 7.35/10, and practiced at home mainly 1–3 times per week or a few times per month, with a smaller group engaging in daily practice.

A thematic qualitative analysis was conducted on 64 open-ended responses collected at post-intervention. The full set of answers is available upon request. Three overarching themes emerged: (a) Perceived benefits, (b) Reported challenges, and (c) Suggestions for improvement.

- Perceived benefits were the most frequently reported and included stress reduction, improved emotional regulation, greater interoceptive awareness, and a stronger body–mind connection. Students described the laboratory as “relaxing,” “therapeutic,” and “the only moment of the day where I could breathe without tension.” Many highlighted learning to recognize bodily sensations and release muscular tension through breath regulation, emphasizing the value of embodied emotional self-regulation.
- Reported challenges included difficulty with breath-focused practices among students with anxiety, initial resistance in entering a meditative state, and discomfort related to practicing in a crowded classroom, although this was reported by a small minority of participants (4). These experiences were described as occasional and often linked to pre-existing anxiety or trauma-related difficulties, particularly during the initial sessions. Importantly, such responses were generally reported as temporary and, in some cases, as improving over time with continued practice. Several students noted that repetitive breathing practices initially increased anxiety before eventually becoming more manageable. Distractions related to noise, movement, or disengaged peers were perceived as limiting concentration.
- Suggestions for improvement focused on increasing exercise variety, expanding available space or reducing group size, and incorporating additional interactive practices (e.g., dyadic or group sensory activities). Some students expressed interest in further exploration of communication, perception, and sensory-based exercises as complements to Qigong.
- Qualitative findings are reported descriptively to illustrate recurrent experiential themes and to identify practical elements relevant for intervention refinement, without aiming to provide a comprehensive interpretative qualitative analysis.

### 3.2. Baseline Comparison Between Completers and Dropouts

Given the high attrition rate, baseline characteristics were compared between participants who completed the study (n = 114) and those who dropped out after the pre-assessment (n = 217) to assess the potential presence of attrition bias (Table S3). No significant differences were observed between completers and dropouts across nearly all sociodemographic and psychological variables. The only exception was the Interpersonal Accuracy Scale (IAS), for which dropouts exhibited slightly higher baseline scores than completers (median [IQR]: 83 [75,76,77,78,79,80,81,82,83,84,85,86,87,88,89,90,91] vs. 81 [72.25–87.50]). The ICQ, DERS, and FFMQ total scores, as well as the PANAS Positive Affect score, were normally distributed and did not differ between groups (all ps > 0.05). For the remaining continuous variables, Mann–Whitney U tests were applied due to clear or borderline deviations from normality, with no statistically significant differences emerging (all ps > 0.05). Likewise, no group differences were found for categorical variables, including age, gender, and previous meditation experience (χ^2^(7) = 5.93, *p* = 0.548; χ^2^(1) = 1.64, *p* = 0.200).

### 3.3. Descriptive Statistics and Normality Tests

Descriptive statistics were computed for all variables, assessed through psychometric questionnaires, and normality was assessed using the Shapiro–Wilk test. Some measures followed a normal distribution (*p* > 0.05), while others deviated significantly from normality (*p* < 0.05), warranting the use of non-parametric tests (Wilcoxon signed rank).

### 3.4. Within-Subjects Analysis

**Figure 1:**
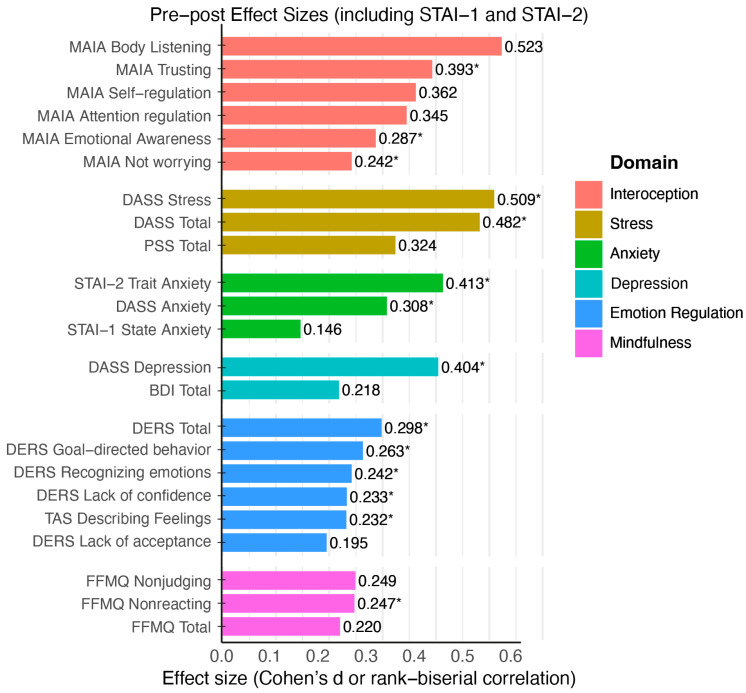
Significant improvements following the intervention: effect sizes (Cohen’s d or rank-biserial correlations) for pre–post changes across psychological variables. Bars are color-coded by psychological domain (interoception, stress, anxiety, depression, emotion regulation, and mindfulness). Effect sizes reflect the magnitude of change following the Qigong-based intervention, with improvements observed in interoception (MAIA), stress (DASS, PSS), depressive symptoms (DASS, BDI), anxiety (STAI-1, STAI-2), and emotion regulation (DERS). Variables are ordered by descending effect size. An asterisk (*) indicates variables analyzed with Wilcoxon signed-rank tests; variables without an asterisk were analyzed using paired-samples *t*-tests.

Paired *t*-tests were conducted to assess differences between pre- and post-intervention scores. Figure 1 displays the results for this analysis.

No significant pre–post changes were observed for the following variables and subscales: DERS–Lack of Awareness; FFMQ–Acting with Awareness, Describing, and Observing; MAIA–Not Distracting; PANAS–Negative Affect and Positive Affect; PSQI; RS; STAI–I; and TAS–Difficulty Identifying Feelings and total score. Full descriptive and inferential statistics for all non-significant results are reported in the Supplementary Materials (Table S4).

### 3.5. Correlations

**Figure 2:**
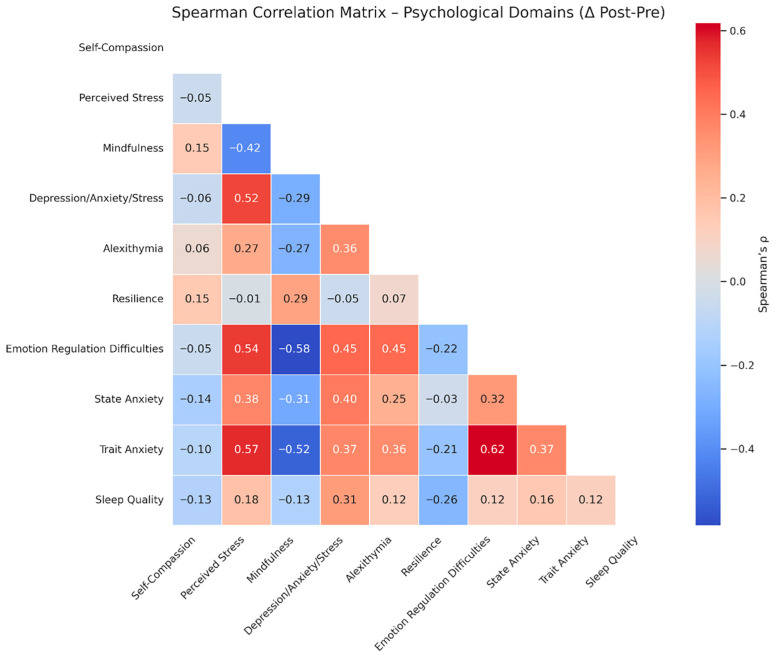
Spearman correlation matrix showing associations between pre–post change scores (Δ) across psychological domains: moderate to strong correlations were observed between changes in mindfulness, perceived stress, anxiety, emotional regulation difficulties, and depressive symptoms. Only post–pre differences (Δ) were used, and domains are labeled for interpretability. Warmer colors indicate stronger positive correlations, while cooler colors indicate negative associations.

Spearman’s rank correlation was used to examine the associations between pre–post change scores (Δ) across some of the psychological variables. Only correlations with |ρ| > 0.30 are reported below. Correlation results are displayed in Figure 2. Correlation analyses were conducted on change scores of variables showing significant pre–post variation, as well as on additional variables of conceptual relevance included for exploratory purposes. Measures that were either non-significant, secondary, or conceptually redundant with core domains were excluded.

Positive correlations:

Perceived Stress (PSS) correlated positively with Trait Anxiety (STAI-2) (*ρ* = 0.57), Emotion Regulation Difficulties (DERS) (*ρ* = 0.54), and Depression, Anxiety, and Stress (DASS) (*ρ* = 0.52).

DASS correlated positively with PSS (*ρ* = 0.52), DERS (*ρ* = 0.45), State Anxiety (STAI-1) (*ρ* = 0.40), and Trait Anxiety (STAI-2) (*ρ* = 0.37).

DERS showed strong positive correlations with Trait Anxiety (STAI-2) (*ρ* = 0.62), PSS (*ρ* = 0.54), DASS (*ρ* = 0.45), and Alexithymia (TAS) (*ρ* = 0.45).

Alexithymia (TAS) was positively correlated with DASS (*ρ* = 0.36), Trait Anxiety (STAI-2) (*ρ* = 0.36), and DERS (*ρ* = 0.45).

State Anxiety (STAI-1) correlated positively with DASS (*ρ* = 0.40), PSS (*ρ* = 0.38), and DERS (*ρ* = 0.32).

Sleep Quality (PSQI) showed a moderate positive correlation with DASS (*ρ* = 0.31).

Negative correlations:

Mindfulness (FFMQ) was negatively correlated with Trait Anxiety (STAI-2) (*ρ* = −0.52), Emotion Regulation Difficulties (DERS) (*ρ* = −0.58), State Anxiety (STAI-1) (*ρ* = −0.31), and Perceived Stress (PSS) (*ρ* = −0.42).

### 3.6. Regression Analyses

To explore whether baseline psychological traits and sociodemographic factors predicted improvements in psychological functioning following the intervention, a series of linear regression analyses were conducted using baseline scores as predictors and the change scores (Δ) of specific psychological variables as outcome measures.

**Figure 3:**
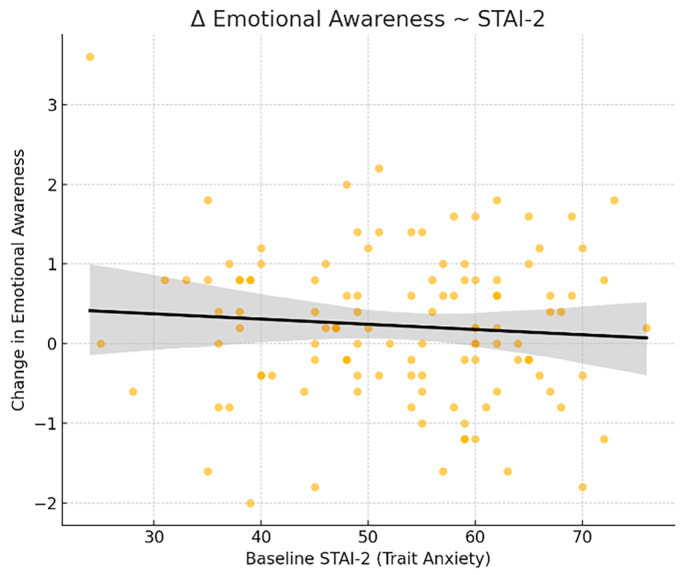
Trait anxiety predicting change in Emotional Awareness: scatterplot showing the relationship between baseline trait anxiety (STAI-2) and changes in the MAIA subscale Emotional Awareness following the intervention. Higher baseline anxiety was associated with greater improvement (β = −0.295, *p* = 0.028).

One regression model revealed that both trait anxiety (STAI-2; Figure 3) and mindfulness (FFMQ) significantly predicted improvement in the MAIA subscale Emotional Awareness. Specifically, higher baseline trait anxiety and lower baseline mindfulness were associated with greater post-intervention improvement in this domain. Trait anxiety negatively predicted the change score (β = −0.295, *p* = 0.028), and similarly, baseline mindfulness also negatively predicted improvement (β = −0.299, *p* = 0.026).

**Figure 4:**
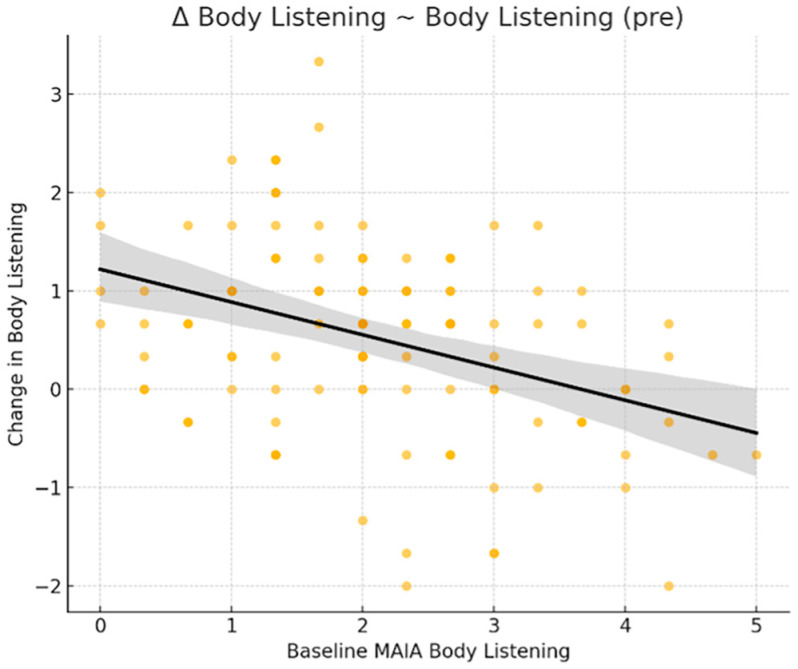
Body Listening predicts its own improvement: scatterplot showing the relationship between baseline scores on the MAIA Body Listening subscale and change scores on the same subscale. Lower initial levels were associated with greater improvements following the intervention (β = −0.376, *p* < 0.001).

Another regression model identified the MAIA subscale Body Listening (Figure 4) as a significant predictor of improvement in the same domain. Lower baseline scores on Body Listening were associated with greater post-intervention gains (β = −0.376, *p* < 0.001).

Sociodemographic variables were also tested as predictors. A significant effect was found for gender, where women showed greater improvement in emotion regulation (DERS) compared to men (β = −0.199, *p* = 0.043).

## 4. Discussion

The present study should be interpreted within the framework of a feasibility and exploratory investigation, aimed at examining the potential psychological effects of a structured Qigong intervention in a university setting. Given the feasibility-oriented design, the assessment of multiple psychological variables, and the absence of predefined primary outcomes or corrections for multiple comparisons, the findings should be considered preliminary rather than confirmatory. In this context, differential improvements observed in participants with lower baseline scores (e.g., Body Listening) may also reflect regression to the mean and therefore require cautious interpretation. Moreover, the high drop-out rate observed in this study warrants careful consideration. Although participation was voluntary, many students were likely unfamiliar with Qigong and may have found the experiential demands of the practice more challenging than initially expected. Embodied exercises involving spontaneous movement, sound emission, and breath-focused attention can elicit discomfort or embarrassment when performed within a large-group setting. In addition, the optional nature of the workshop and competing academic demands may have contributed to reduced adherence over time. We will discuss this point further in Section 4.2.

Turning to the quantitative findings, the present study revealed significant improvements across several key dimensions of interoceptive awareness, as assessed by the MAIA. Participants reported increased Body Listening, reflecting a greater tendency to attend to bodily cues as sources of guidance and information, as well as higher Trusting, indicating a strengthened perception of the body as a safe and reliable reference. Improvements were also observed in Self-Regulation, Attention Regulation, and Emotional Awareness, together with reductions in Not Worrying, suggesting a decreased tendency to experience distress in response to bodily sensations. Overall, these changes point to a more adaptive and functional relationship with bodily experience. Such findings are concordant with previous evidence indicating that mind–body practices enhance interoceptive functioning and extend recent work in this area (e.g., Chang et al. [55]). As no control condition was included, these results should be interpreted cautiously.

From a mechanistic perspective, improvements across interoceptive subcomponents are particularly relevant because interoceptive awareness is increasingly conceptualized as a foundation for emotional functioning and self-regulation. Enhanced body listening, trust in bodily sensations, and the regulatory use of interoceptive cues have been identified as key pathways through which contemplative and body-based interventions may reduce stress vulnerability and promote psychological well-being. In the present study, gains in interoceptive awareness were accompanied by parallel improvements in emotion regulation, as reflected in reductions across multiple DERS dimensions, including difficulties in goal-directed behavior, emotional awareness, non-acceptance of emotional responses, and limited access to regulation strategies.

Qigong, as a movement-based contemplative practice, has been associated with a state of relaxed alertness and increased vagal tone, reflecting a dynamic balance between parasympathetic and sympathetic activity. In line with models emphasizing the interaction between top-down and bottom-up regulatory pathways [9], the integration of voluntary and spontaneous movements in the present protocol may support complementary mechanisms of emotion regulation. Specifically, voluntary movements and sustained attentional engagement may recruit top-down cognitive control processes, whereas spontaneous movements may enhance bottom-up proprioceptive and interoceptive signaling, fostering more embodied and sensory-driven forms of regulation [85]. Given the overlap between neural substrates involved in visceral perception, affective awareness, and regulatory processes [86,87], as well as the established links between bodily awareness, emotional processing, and psychopathology [3,26,54], enhanced interoceptive capacity may represent one pathway through which Qigong contributes to changes in emotional functioning.

In line with this interpretation, although total alexithymia scores did not show significant changes, specific components related to externally oriented thinking and difficulties in describing feelings improved over time. This pattern suggests a selective effect on aspects of emotional awareness and symbolic elaboration, rather than on global alexithymic traits, and may be linked with the observed changes in interoceptive awareness and emotion regulation.

The correlational analyses support a systemic view of the observed changes. As shown in Figure 2, moderate-to-strong positive associations emerged among reductions in perceived stress, trait anxiety, emotion regulation difficulties, and depressive symptoms (e.g., ρ = 0.57 between PSS and STAI-II; ρ = 0.54 between PSS and DERS; ρ = 0.62 between DERS and STAI-II), suggesting that improvements across these domains co-occurred within a coherent pattern of psychological change. Notably, the reduction observed in trait anxiety, in the absence of significant changes in state anxiety, indicates that the intervention may have preferentially influenced more stable dispositional patterns rather than transient affective states. This finding aligns with the characteristics of Qigong practice, which may foster changes in more enduring regulatory processes.

In contrast, increases in mindfulness were negatively associated with perceived stress, trait anxiety, and emotion regulation difficulties, highlighting its potential protective role within this broader network of changes. Associations involving alexithymia and emotion regulation difficulties are also compatible with previous research linking personality traits, distress proneness, and regulatory vulnerabilities across clinical and non-clinical populations [37,49,87,88,89,90,91,92,93].

At the same time, several assessed variables did not show significant pre–post changes, including facets of dispositional mindfulness primarily related to attentional monitoring (e.g., Observing, Describing, Acting with Awareness), general affective states (PANAS), and the MAIA subscale Not Distracting. The absence of changes in positive and negative affect supports the interpretation that the intervention primarily targeted interoceptive and regulatory processes, rather than producing generalized shifts in mood. Taken together, this pattern suggests that the intervention may have preferentially influenced embodied, bottom-up regulatory and relational processes—such as the capacity to attend to bodily signals, regulate internal states, and experience the body as a trustworthy source of information—rather than top-down attentional control or trait-like affective dispositions. In a non-clinical sample, these latter dimensions may be relatively stable and less responsive to short-term interventions, or may require longer practice duration to show measurable change.

Baseline individual differences further moderate outcomes. Participants with higher trait anxiety and lower baseline mindfulness showed greater gains in Emotional Awareness, whereas those with lower initial Body Listening exhibited the largest improvements in this domain. A modest gender effect was also observed, with females showing greater improvements in emotion regulation. These findings suggest that individuals with greater initial interoceptive difficulties or emotional vulnerability may particularly benefit from Qigong practice, potentially because it targets regulatory processes that are more compromised at baseline. While this pattern suggests greater responsiveness among participants with lower baseline levels, regression to the mean cannot be excluded as an alternative explanation.

Qualitative findings complemented the quantitative results. Most participants, despite having little or no prior experience with meditation or Qigong, described the intervention as relaxing and helpful for increasing awareness of bodily tension and breathing patterns. However, a little part of the students, particularly those with higher anxiety—reported initial difficulties with breath-focused practices, and others noted discomfort related to practicing in a crowded classroom. These observations suggest that engagement in Qigong may depend on how safely and comfortably individuals can attend to bodily sensations, especially when interoceptive awareness is initially limited.

From a clinical perspective, Qigong appears promising as a low-cost intervention for university students experiencing elevated stress, emotion regulation difficulties, interoceptive impairments, or subthreshold symptoms of anxiety, depression, and alexithymia. Improvements in Trusting and Not Worrying may foster a more compassionate bodily self-relation, which is considered central in trauma-informed approaches [57,63]. More broadly, these findings align with models conceptualizing interoceptive dysfunction as a transdiagnostic marker [94] and emotion regulation as a key resilience factor [15]. The integration of top-down and bottom-up techniques may therefore be particularly suitable for academic mental health programs. Interpretation of these findings requires consideration of several methodological limitations, discussed in the following section.

### 4.1. Limitations

Several limitations should be acknowledged. First, the study did not include a control group; therefore, causal inferences regarding the effects of the Qigong intervention cannot be drawn, and observed changes may partly reflect nonspecific factors such as group participation, the academic context, or regression to the mean. Second, the high dropout rate between pre- and post-assessment (approximately 65%) substantially reduced the final sample size and represents a relevant source of potential bias. Although baseline comparisons between completers and dropouts showed comparable sociodemographic and psychological characteristics, the possibility of bias related to unmeasured or latent factors (e.g., motivation, distress tolerance, or attitudes toward body-focused practices) cannot be excluded. Third, classroom constraints limited the range of movements and restricted the exploration of spontaneous movement, which may elicit emotionally salient responses requiring appropriate containment. Moreover, the use of self-report measures means that results pertain specifically to interoceptive awareness, as a subjective and appraisal-based construct, and not to interoceptive accuracy or objective physiological indices.

Finally, the absence of follow-up assessments prevents conclusions regarding the durability of the observed effects over time. Despite these limitations, the study provides preliminary evidence from a large non-clinical sample and represents one of the few evaluations of Qigong with spontaneous movement conducted in a university setting.

### 4.2. Lessons Learned and Implications for Future Research

As this Qigong program was implemented for the first time within an Italian university setting, several methodological lessons can be drawn to inform the design of a more definitive trial.

First, the physical setting of the intervention should be reconsidered in light of the difficulties reported by participants. In-person sessions could be conducted in larger spaces such as sports halls or outdoor environments, in order to reduce crowding and minimize discomfort, particularly among students with elevated anxiety or attentional difficulties. In addition, the use of digital communication tools (e.g., group chats or dedicated online platforms) may facilitate real-time support and clarification regarding daily home practice, thereby enhancing engagement and adherence.

Second, given that Qigong is still relatively unfamiliar to most Italian students, future studies would benefit from the inclusion of preliminary educational sessions prior to the start of the intervention. Providing clear information about the nature of Qigong practice, including the presence of both voluntary and spontaneous movements, may help align participants’ expectations and reduce early dropout. In the present study, some attrition may have been related to discomfort or embarrassment associated with spontaneous movements in a large-group context; preparatory meetings could foster a more informed and receptive attitude toward the practice.

Third, a more definitive trial should rely on a structured and adequately resourced intervention framework. The management of large groups practicing embodied and emotionally salient exercises requires the presence of trained facilitators capable of providing containment, guidance, and individual support when needed. Such an organizational structure may allow participants to engage more deeply with Qigong-related mind–body states and to integrate these experiences into daily life.

With regard to outcomes, future studies may extend assessment beyond psychological well-being to include longer-term indicators of academic functioning, such as perceived academic stress, concentration, engagement, or academic performance. These outcomes may be particularly relevant once sustained practice allows for more stable integration of self-regulatory skills.

Finally, a definitive trial should include an appropriate control condition. A credible control group could be drawn from the same student population to ensure comparable baseline characteristics. From an ethical perspective, a waitlist control design may be particularly suitable in a university context, although its implementation may present practical challenges. Alternative active control conditions could also be considered in order to better isolate the specific contribution of Qigong practice.

## 5. Conclusions

This study provides preliminary evidence suggesting that a structured Qigong program may be associated with improvements in interoceptive awareness, emotion regulation, and psychological well-being in university students. These effects may result from the combined use of static and dynamic exercises that engage both top-down and bottom-up processes. Benefits were particularly pronounced in students with lower baseline interoceptive ability or higher trait anxiety. Given its affordability, ease of implementation, and broad psychological benefits, Qigong represents a promising preventive tool to support mental health and resilience in educational settings. Further research using controlled and methodologically rigorous designs is needed to assess the stability of these effects over time, incorporate physiological measures, and clarify the specific therapeutic contribution of spontaneous movement within Qigong practice.

## Data Availability

The data supporting the findings of this study are not publicly available due to privacy restrictions but can be provided by the corresponding author upon reasonable request.

## Supplementary Materials

The following supporting information can be downloaded at: https://www.mdpi.com/article/10.3390/healthcare14020202/s1, Table S1: Program of the twelve intervention sessions for the Qigong workshop. Table S2: Demographic variables collected prior to the intervention. Table S3: Baseline characteristics of completers and dropouts. Table S4: Results of paired-sample tests evaluating pre–post changes across all variables.
