# Supplementary material for: Mind the Motion: Feasibility and Effects of a Qigong Intervention on Interoception and Well-Being in Young Adults
Source: Healthcare (Basel). 2026 Jan 13;14(2):202. doi: 10.3390/healthcare14020202 (PMC12841306; doi:10.3390/healthcare14020202)
Supplement: Supplementary file 1 [file healthcare-14-00202-s001.zip › healthcare-4030434-supplementary.pdf]

Supplementary Material

Historical and Conceptual Background of Qigong

Qigong refers to a broad and heterogeneous family of mind–body practices that developed over centuries within different strands of Chinese culture, including Daoist self-cultivation traditions, medical practices, and martial arts. While techniques resembling what is now called Qigong can be traced back to early Daoist and medical texts, these practices were historically embedded within specific philosophical, cosmological, and experiential frameworks rather than defined as a unified system [1]. In particular, Daoist internal alchemy traditions (Neidan) emphasized the regulation of breath, posture, attention, and internal sensations as means of cultivating bodily vitality and mental clarity, privileging internal transformation over external or chemical methods [1].

The term Qigong (氣功), however, is a relatively recent construct. It gained widespread usage during the mid-twentieth century in the context of political, scientific, and institutional reforms in the People's Republic of China. During this period, diverse traditional practices were selectively systematized, standardized, and reframed within a modern, secular discourse aimed at aligning them with biomedical, physiological, and psychological models [2]. This process facilitated the integration of Qigong into hospitals, rehabilitation programs, and public health initiatives, while also distancing it from explicitly religious or metaphysical interpretations.

As a result of this historical transformation, contemporary Qigong represents a hybrid practice situated at the intersection of traditional cultivation methods and modern health-oriented frameworks. Although historical lineages and styles remain highly diverse, modern Qigong interventions are typically characterized by structured protocols involving gentle, repetitive movements, postural regulation, breath control, and attentional training [3]. These features make Qigong particularly adaptable to empirical investigation and implementation in clinical and non-clinical contexts, including educational settings.

Importantly, this process of modernization does not imply conceptual uniformity or theoretical consensus. Rather, Qigong encompasses a spectrum of practices that differ in emphasis on movement, stillness, volitional control, and spontaneous bodily processes. From a research perspective, this heterogeneity underscores the importance of clearly specifying the form of Qigong employed and the mechanisms of interest, rather than treating Qigong as a monolithic intervention. The present study adopts this approach by focusing on a structured Neidan-inspired protocol designed to integrate contemplative attention, movement, and embodied awareness within a university-based intervention.

Table S1: Program of the twelve intervention sessions for the Qigong workshop

Week #1	A. **Settling In**<br>Allow participants to get comfortable in a seated position.<br>B. **Introduction to Qigong**<br>Present the agendas and contents related to the intervention sessions and home daily assignment.<br>C. **Mindfulness Awareness Experience**<br>In a seated or standing position, participants are guided to bring awareness to the breath, sensations, emotions, thoughts, and to cultivate the ability to be aware of whatever arises in the present moment experience.<br>D. **Body Scan with Focus on Sensations**<br>In a seated or standing position, participants are invited to observe their sensations by either focusing on precise body districts or through an open monitoring.
Homework Assignments	● Repetitive Movements<br>● Body Scan
Week #2	A. **Settling In**<br>Allow participants to get comfortable in a seated position.<br>B. **Sitting Breathwork**<br>Participants are invited to observe and explore the breath at the upper chest, the belly, and the whole body. Afterwards, facilitators lead them to directional breathing focusing on upper and lower diaphragm.<br>C. **Repetitive Movements**<br>Rolling shoulders forward and backward, turn head in all directions, circling wrists forward and backward, body shaking, body swing, body bouncing.<br>D. **Sitting Meditation**<br>In a seated position, participants are invited to focus their attention on the breath, to be aware when the attention disconnects from the breath without judgment, and to refocus on the breath.
Homework Assignments	● Breathwork<br>● Repetitive Movements<br>● Sitting Meditation
Week #3	A. **Settling In**<br>Allow participants to get comfortable in a seated position.<br>B. **Body Scan with Focus on Sensations**<br>In a seated or standing position, participants are invited to observe their sensations by either focusing on precise body districts or through an open monitoring. Sound emission: in a seated or standing position, while exhaling, participants are told to pronounce a sound, paying special attention to the vibrations that propagate throughout their bodies.<br>C. **Repetitive Movements**<br>Pull whole body heeling up, shaking out arms and legs, body brushing, body shaking, body swing, body bouncing, jumping.<br>D. **Standing Meditation (Zhan Zhuang)**<br>Participants are instructed to relax, assume, and keep standing in a particular posture, characterized by legs aligned with the shoulders' width, slightly bent knees, dropped elbows, hands clasped in front of the head, eyes closed or looking forward. Breathing should be deep, long, and even.
Homework Assignments	● Body Scan<br>● Repetitive Movements<br>● Standing Meditation
Week #4	A. **Settling In**<br>Allow participants to get comfortable in a seated position.<br>B. **Sitting Breathwork**<br>Participants are taught to direct their attention to the lower abdomen, bring the hands with palms facing up on the knees, and breath in, trying to direct the breath around the internal bodily orbit. This orbit is described as an imaginary energy channel wherein the energy flows from the eyes to the lower abdomen, then up again to the mouth. Participants are invited to keep calm and collected, as well as to use the nose to inhale and the mouth to exhale.<br>C. **Repetitive Movements**<br>Turn waist clockwise and counterclockwise, opening and closing of the spine, lifting and stretching knee, body shaking, body swing, body bouncing, jumping.<br>D. **Standing Meditation (Zhan Zhuang)**<br>Participants are instructed to relax, assume, and keep standing in a particular posture, characterized by legs aligned with the shoulders' width, slightly bent knees, dropped elbows, hands clasped in front of the head, eyes closed or looking forward. Breathing should be deep, long, and even.
Homework Assignments	● Breathwork<br>● Repetitive Movements<br>● Standing Meditation
Week #5	A. **Settling In**<br>Allow participants to get comfortable in a seated position.<br>B. **Mindfulness Awareness Experience**

	In a seated or standing position, participants are guided to bring awareness to the breath, sensations, emotions, thoughts, and to cultivate the ability to be aware of whatever arises in the present moment experience. Sound emission: in a seated or standing position, while exhaling, participants are told to pronounce a sound, paying special attention to the vibrations that propagate throughout their bodies.<br>C. **Repetitive Movements**<br>Stretching ankles and circling, balanced oscillations, spiraling movements, body bouncing, spontaneous movements.<br>D. **Sitting Meditation**<br>In a seated position, participants are invited to focus their attention on the breath, to be aware when the attention disconnects from the breath without judgment, and to refocus on the breath.
Homework Assignments	● Sound Emission<br>● Repetitive Movements<br>● Sitting Meditation
Week #6	A. **Settling In**<br>Allow participants to get comfortable in a seated position.<br>B. **Standing Breathwork**<br>Participants are instructed to relax and assume a particular posture, characterized by legs aligned with the shoulders' width, dropped elbows, eyes closed or looking forward. While inhaling, both hands are raised up to the top of the head, trying to lean the whole body backward. While exhaling, the hands (palms facing down) drop on the side of the body.<br>C. **Repetitive Movements**<br>Rolling shoulders forward and backward, turn head in all directions, circling wrists forward and backward, body shaking, body swing, body bouncing.<br>D. **Standing Meditation (Zhan Zhuang)**<br>Participants are instructed to relax, assume, and keep standing in a particular posture, characterized by legs aligned with the shoulders' width, slightly bent knees, dropped elbows, hands clasped in front of the head, eyes closed or looking forward. Breathing should be deep, long, and even.
Homework Assignments	● Breathwork<br>● Repetitive Movements<br>● Standing Meditation
Week #7	A. **Settling In**<br>Allow participants to get comfortable in a seated position.<br>B. **Mindfulness Awareness Experience**<br>In a seated or standing position, participants are guided to bring awareness to the breath, sensations, emotions, thoughts, and to cultivate the ability to be aware of whatever arises in the present moment experience.<br>C. **Repetitive Movements**<br>Body shaking, body swing, body bouncing, spontaneous movements.<br>D. **Standing Meditation (Zhan Zhuang)**<br>Participants are instructed to relax, assume, and keep standing in a particular posture, characterized by legs aligned with the shoulders' width, slightly bent knees, dropped elbows, hands clasped in front of the head, eyes closed or looking forward. Breathing should be deep, long, and even.
Homework Assignments	● Mindfulness<br>● Repetitive Movements<br>● Standing Meditation
Week #8	A. **Settling In**<br>Allow participants to get comfortable in a seated position.<br>B. **Sitting Breathwork**<br>Participants are taught to direct their attention to the lower abdomen, bring the hands with palms facing up on the knees, and breath in, trying to direct the breath around the internal bodily orbit. This orbit is described as an imaginary energy channel wherein the energy flows from the eyes to the lower abdomen, then up again to the mouth. Participants are invited to keep calm and collected, as well as to use the nose to inhale and the mouth to exhale.<br>C. **Repetitive Movements**<br>Pull whole body heeling up, shaking out arms and legs, body brushing, body shaking, body swing, body bouncing, jumping.<br>D. **Sitting Meditation**<br>In a seated position, participants are invited to focus their attention on the breath, to be aware when the attention disconnects from the breath without judgment, and to refocus on the breath.
Homework Assignments	● Breathwork<br>● Repetitive Movements<br>● Sitting Meditation
Week #9	A. **Settling In**<br>Allow participants to get comfortable in a seated position.<br>B. **Standing Breathwork**<br>Participants are instructed to relax and assume a particular posture, characterized by legs aligned with the shoulders' width, dropped elbows, eyes closed or looking forward. While inhaling, both hands are raised up to the top of the head, trying to lean the whole body backward. While exhaling, the hands (palms facing down) drop on the side of the body.

	C. **Repetitive Movements**<br>Turn waist clockwise and counterclockwise, opening and closing of the spine, lifting and stretching knee, body shaking, body swing, body bouncing.<br>D. **Standing Meditation (Zhan Zhuang)**<br>Participants are instructed to relax, assume, and keep standing in a particular posture, characterized by legs aligned with the shoulders' width, slightly bent knees, dropped elbows, hands clasped in front of the head, eyes closed or looking forward. Breathing should be deep, long, and even.
Homework Assignments	● Breathwork<br>● Repetitive Movements<br>● Standing Breathwork
Week #10	**A. Settling In**<br>Allow participants to get comfortable in a seated position.<br>B. **Sitting Breathwork**<br>Participants are invited to observe and explore the breath at the upper chest, the belly, and the whole body. Afterwards, facilitators lead them to directional breathing focusing on upper and lower diaphragm. Sound emission: in a seated or standing position, while exhaling, participants are told to pronounce a sound, paying special attention to the vibrations that propagate throughout their bodies.<br>C. **Repetitive Movements**<br>Stretching ankles and circling, balanced oscillations, spiraling movements, spontaneous movements.<br>D. **Standing Meditation (Zhan Zhuang)**<br>Participants are instructed to relax, assume, and keep standing in a particular posture, characterized by legs aligned with the shoulders' width, slightly bent knees, dropped elbows, hands clasped in front of the head, eyes closed or looking forward. Breathing should be deep, long, and even.
Homework Assignments	● Sound Emission<br>● Repetitive Movements<br>● Standing Meditation
Week #11	A. **Settling In**<br>Allow participants to get comfortable in a seated position.<br>B. **Standing Breathwork**<br>Participants are instructed to relax and assume a particular posture, characterized by legs aligned with the shoulders' width, dropped elbows, eyes closed or looking forward. While inhaling, both hands are raised up to the top of the head, trying to lean the whole body backward. While exhaling, the hands (palms facing down) drop on the side of the body.<br>C. **Repetitive Movements**<br>Rolling shoulders forward and backward, turn head in all directions, circling wrists forward and backward, body shaking, body swing, body bouncing, jumping.<br>D. **Sitting Meditation**<br>In a seated position, participants are invited to focus their attention on the breath, to be aware when the attention disconnects from the breath without judgment, and to refocus on the breath.
Homework Assignments	● Breathwork<br>● Repetitive Movements<br>● Sitting Meditation
Week #12	A. **Settling In**<br>Allow participants to get comfortable in a seated position.<br>B. **Mindfulness Awareness Experience**<br>In a seated or standing position, participants are guided to bring awareness to the breath, sensations, emotions, thoughts, and to cultivate the ability to be aware of whatever arises in the present moment experience.<br>C. **Repetitive Movements**<br>Pull whole body heeling up, shaking out arms and legs, body brushing, body shaking, body swing, body bouncing, jumping.<br>D. **Standing Meditation (Zhan Zhuang)**<br>Participants are instructed to relax, assume, and keep standing in a particular posture, characterized by legs aligned with the shoulders' width, slightly bent knees, dropped elbows, hands clasped in front of the head, eyes closed or looking forward. Breathing should be deep, long, and even.
Homework Assignments	● Mindfulness<br>● Repetitive Movements<br>● Standing Meditation

Table S2. Demographic variables collected prior to the intervention. Table S2 summarizes the demographic and background information collected at baseline.

	Variable	Type/Format	Response options
PRE-TEST	**Age**	Continuous (years)	Numeric value
	**Gender**	Categorical	Female; Male; Other (i.e. Non-binary; Gender fluid)
	**Marital status**	Categorical	Single; Married/Co-habiting; Remarried; Separated/Divorced; Widowed
	**Educational level**	Categorical	High school diploma; Bachelor's degree; PhD; Postgraduate diploma (e.g., Master's); Other
	**Employment status**	Categorical	Full-time student; Student with full-time job; Student with part-time job; Student with occasional job; Other
	**Previous meditation experience**	Categorical + Open-ended	Yes/No + Type (e.g., mindfulness, vipassana, transcendental)
	**Meditation frequency**	Categorical	Never; Daily; 1–2 times/week; Monthly; <10 times/year
	**Previous Qi Gong experience**	Categorical	Yes/No
	**Qi Gong frequency**	Categorical	Never; Daily; 1–2 times/week; Monthly; <10 times/year
	**Intention to participate in the Qi Gong course**	Dichotomous	Yes/No
POST-TEST	**Participation in the Qi Gong course**	Dichotomous	Yes/No
	**Overall interest in the course**	11-point rating (0–10)	0 = no interest; 10 = maximal interest
	**Class attendance**	11-point rating (0–10)	0 = none; 10 = full attendance
	**Frequency of home practice**	Categorical	Never; Daily; 4–6/week; 1–3/week; Few times/month; Other
	**Frequency of in-class participation**	Categorical	Never; 1–4 times; 5–9 times; Most of the time; Always; Other
	**Open-ended feedback on course experience**	Open text	Strengths, weaknesses, learning, suggestions

Table S3. Baseline Characteristics of Completers and Dropouts

Variables	**Completers** (n=114)	**Dropouts** (n=217)	p-value
**Age**	20 [19–21]	20 [19–21]	.997
**Gender**	F=86 (75.44%)<br>M=26 (22.81%)<br>Other= 2 (1.75%)	F=165 (76.04%)<br>M=49 (22.58%)<br>Other=3 (1.38%)	.964
**Meditation experience (yes/no)**	Yes=34 (29.82%)<br>No=80 (70.18%)	Yes=80 (36.87%)<br>No=137 (63.13%)	.200
**FFMQ - Total**	112.5 (17.70)	114.1 (16.15)	.292
**PSS**	22 [18–27.75]	24 [18–30]	.180
**IAS**	81 [72.25–87.50]	83 [75–91]	.041*
**ICQ**	54.81 (7.64)	55.85 (7.97)	.254
**STAI-I**	46.50 [38.25–57]	49 [38–57]	.729
**STAI-II**	55 [45–61.75]	54 [45–62]	.909
**MAIA - Body Listening**	2 [1.33–2.67]	2 [1.33–3.0]	.954
**DASS**	23 [12–37.50]	23 [13–34]	.625
**DERS - Total**	93.74 (24.37)	94.54 (25.76)	.783
**BDI**	15 [9–25]	15 [8–25]	.852
**PANAS - Positive Affect**	27.34 (8.54)	26.76 (8.27)	.551
**PANAS - Negative Affect**	19 [13–27]	19 [13–29]	.720
**PSQI**	6.5 [5–9.25]	6 [4–9]	.366
**RS**	64 [54–73.75]	66 [56–73]	.479
**SCS**	2.67 [2.28–3.31]	2.7 [2.25–3.067]	.969
**TAS**	51 [42.25–58.75]	52 [42–60]	.339

Note. Consistent with the multidimensional conceptualization of interoception, MAIA scores were examined at the subscale level. Among the MAIA subscales, Body Listening was selected as it most directly captures the tendency to actively attend to and use bodily signals to guide cognitive and emotional processes. FFMQ, DERS, PANAS - Positive Affect were normally distributed and are therefore reported as Mean (SD) and compared using an independent-samples t-test. All other continuous variables showed non-normal or mixed distribution patterns between groups, and were consequently reported as Median [IQR] and compared using Mann–Whitney U test. Categorical variables were compared using chi-square test as this test is appropriate for assessing differences in frequency distributions between independent groups.

Table S4. Results of paired-sample tests evaluating pre–post changes across all variables. Table S4 provides a summary of both significant and non-significant results across all measures.

Variable	Subscale	Test	Statistic (t/z)	p-value	Effect Size	Confidence Interval (CI)
**BDI**		1	2.332	**.021***	0.218	[0.032, 0.404]
**DASS – Total**		2	4.310	**<.001***	0.482	[0.297, 0.632]
	DASS - Anxiety	2	2.686	**.007***	0.308	[0.093, 0.495]
	DASS - Depression	2	3.543	**<.001***	0.404	[0.202, 0.573]
	DASS – Stress	2	4.348	**<.001***	0.509	[0.320, 0.658]
**DERS – Total**		2	2.722	**.007***	0.298	[0.092, 0.479]
	DERS – Difficulties in Goal-directed behaviour	2	2.321	**.020***	0.263	[0.047, 0.456]
	DERS – Lack of Acceptance	1	2.086	**.039***	0.195	[0.010, 0.380]
	DERS – Lack of Awareness	1	-0.620	.537	-0.058	[-0.242, 0.126]
	DERS – Lack of Clarity	2	2.184	**.029***	0.242	[0.030, 0.434]
	DERS – Lack of Control	1	2.362	**.020***	0.221	[0.035, 0.407]
	DERS – Lack of Strategies	2	2.041	**.041***	0.233	[0.014, 0.431]
**FFMQ - Total**		1	-2.346	**.021***	-0.220	[-0.405, -0.033]
	FFMQ - Acting with awareness	1	-0.239	.811	-0.022	[-0.206, 0.161]
	FFMQ – Describing	1	0.204	.838	0.019	[-0.164, 0.203]
	FFMQ – Non Judging	1	-2.665	**.009***	-0249	[-0.435, -0.062]
	FFMQ – Non Reacting	2	-2.211	**.027***	-0.247	[-0.440, -0.033]
	FFMQ - Observing	1	-0.690	.492	-0.065	[-0.248, 0.119]
**MAIA – Attention Regulation**		1	-3.685	**<.001***	-0.345	[-0.533, -0.155]
**MAIA – Body Listening**		1	-5.583	**<.001***	-0.523	[-0.718, -0.326]
**MAIA – Emotional Awareness**		2	-2.542	**.011***	-0.287	[-0.475, -0.074]
**MAIA – Not Distracting**		1	0.842	.402	0.079	[-0.105, 0.263]

**MAIA – Noticing**		1	-2.302	**.023***	-0.216	[-0.401, -0.029]
**MAIA – Not Worrying**		1	-2.580	**.011***	-0.242	[-0.427, -0.055]
**MAIA – Self Regulation**		1	-3.870	**<.001***	-0.362	[-0.551, -0.172]
**MAIA – Trusting**		2	-3.216	**.001***	-0.393	[-0.574, -0.174]
**PANAS – Negative Affect**		2	0.594	.553	0.067	[-0.154, 0.282]
**PANAS – Positive Affect**		1	0.334	.739	0.031	-0.152, 0.215]
**PSQI**		2	-0.094	.926	-0.011	[-0.246, 0.224]
**PSS**		1	3.455	**<.001***	0.324	[0.135, 0.511]
**RS**		1	1.427	.156	0.134	[-0.051, 0.318]
**STAI-I**		1	1.562	.121	0.146	[-0.039, 0.331]
**STAI-II**		2	3.688	**<.001***	0.413	[0.216, 0.577]
**TAS - Total**		1	1.052	.295	0.099	[-0.086, 0.282]
	TAS – Difficulty in Describing Feelings	2	2.045	**.041***	0.232	[0.014, 0.429]
	TAS – Difficulty in Identifying Feelings	2	1.793	.072	0.209	[-0.016, 0.413]
	TAS – Externally Oriented Thinking	1	-2.897	**.005***	-0.271	[-0.458, -0.084]

Note: For each measure, the table reports the statistical test used (Student's t-test= 1 or Wilcoxon signed-rank test= 2), the test statistic (t or Z), the p-value, the effect size, and the 95% confidence interval.
